# Ecological consequences of invasion across the freshwater–marine transition in a warming world

**DOI:** 10.1002/ece3.3652

**Published:** 2018-01-11

**Authors:** Daniel Crespo, Martin Solan, Sara Leston, Miguel A. Pardal, Marina Dolbeth

**Affiliations:** ^1^ Department of Life Sciences Centre for Functional Ecology—CFE University of Coimbra Coimbra Portugal; ^2^ Ocean and Earth Science National Oceanography Centre Southampton University of Southampton Southampton UK; ^3^ CNC—Center for Neuroscience and Cell Biology Pharmacy Faculty University of Coimbra Coimbra Portugal; ^4^ MARE‐Marine and Environmental Sciences Centre IPL, Escola Superior de Turismo e Tecnologia do Mar Peniche Portugal; ^5^ CIIMAR—Interdisciplinary Centre of Marine and Environmental Research of the University of Porto Novo Edifício do Terminal de Cruzeiros do Porto de Leixões Matosinhos Portugal

**Keywords:** coastal transition zone, ecosystem functioning, invasive species, nonindigenous species, refugia, warming

## Abstract

The freshwater–marine transition that characterizes an estuarine system can provide multiple entry options for invading species, yet the relative importance of this gradient in determining the functional contribution of invading species has received little attention. The ecological consequences of species invasion are routinely evaluated within a freshwater versus marine context, even though many invasive species can inhabit a wide range of salinities. We investigate the functional consequences of different sizes of *Corbicula fluminea*—an invasive species able to adapt to a wide range of temperatures and salinity—across the freshwater–marine transition in the presence versus absence of warming. Specifically, we characterize how *C. fluminea* affect fluid and particle transport, important processes in mediating nutrient cycling (NH_4_‐N, NO_3_‐N, PO_4_‐P). Results showed that sediment particle reworking (bioturbation) tends to be influenced by size and to a lesser extent, temperature and salinity; nutrient concentrations are influenced by different interactions between all variables (salinity, temperature, and size class). Our findings demonstrate the highly context‐dependent nature of the ecosystem consequences of invasion and highlight the potential for species to simultaneously occupy multiple components of an ecosystem. Recognizing of this aspect of invasibility is fundamental to management and conservation efforts, particularly as freshwater and marine systems tend to be compartmentalized rather than be treated as a contiguous unit. We conclude that more comprehensive appreciation of the distribution of invasive species across adjacent habitats and different seasons is urgently needed to allow the true extent of biological introductions, and their ecological consequences, to be fully realized.

## 1 INTRODUCTION

Estuaries are transitional areas that face cyclic variations in physico‐chemical and biotic conditions. These interface areas are at the forefront of global ecological changes (Grilo, Cardoso, Dolbeth, Bordalo, & Pardal, 2011; IPCC, 2013; Rabalais, Turner, Díaz, & Justić, 2009) and are particularly prone to invasion by nonindigenous species because of their proximity to human populations (Cohen, Small, Mellinger, Gallup, & Sachs, 1997) that introduce major vectors for introductions (Crespo, Dolbeth, Leston, Sousa, & Pardal, 2015; Gallardo, Clavero, Sánchez, & Vilà, 2016; Williams & Grosholz, 2008). Simultaneously, increasing sea‐surface temperatures, rising sea levels, increasing atmospheric CO_2_ concentrations, and ocean acidification are already altering coastal and marine habitats (Kroeker, Kordas, Crim, & Singh, 2010; Levitus, Antonov, Boyer, & Stephens, 2000; Parmesan & Yohe, 2003) which may, in turn, further modify the likelihood and rate of the introduction of nonindigenous species (Rahel & Olden, 2008; Stachowicz, Terwin, Whitlatch, & Osman, 2002; Williams & Grosholz, 2008).

Estuaries are highly productive habitats (Dolbeth et al., 2011; Hicks et al., 2011; Kennish, 2002) and functionally important areas (e.g., Sousa, Lillebø, Pardal, & Caçador, 2010; Sousa, Lillebø, Risgaard‐Petersen, Pardal, & Caçador, 2012). They are generally characterized by low diversity, constrained by local environmental conditions (Dolbeth et al., 2011; Hicks et al., 2011; Kennish, 2002), such that the introduction of new species that have different traits to the recipient community can have a disproportionate effect on the functioning of the ecosystem (Darrigran & Damborenea, 2011; Simberloff et al., 2013; Stachowicz & Byrnes, 2006). In this respect, nonindigenous invasive species (NIS) may shift the composition of native communities or otherwise propagate ecological impacts throughout the food web and generate associated positive or negative effects on ecosystem functioning. These, in turn, can be alleviated or exacerbated by other factors such as climate change, nutrient loading, land use alteration, and several other anthropogenic‐induced changes (Stachowicz et al., 2002; Strayer & Hillebrand, 2012).

Ecological modifications due to invasive events are generally described as having negative effects on the resident communities and ecosystem processes (e.g., biodiversity loss, biofouling), but positive effects have also been described (e.g., local economy, improvement of water quality) (Dolbeth, Cusson, Sousa, & Pardal, 2012; Katsanevakis et al., 2014; Rosa et al., 2011). Nevertheless, estuaries as transitional habitats face species introductions from freshwater and/or marine sources, exacerbated by the gradient in environmental conditions that has the potential to provide multiple entry points, generate refugia opportunities, and influence source–sink dynamics that affect the long‐term presence and exchange of individuals between populations (Heinrichs, Lawler, & Schumaker, 2016).

Most work on biological invasions focus on specific habitats and how the introduced species interact with native populations and communities (e.g., Ilarri et al., 2012; Simberloff et al., 2013; Strayer & Hillebrand, 2012; Williams & Grosholz, 2008). However, in most cases, it is difficult to establish cause–effect relationships without experimental studies (Grosholz & Ruiz, 2009). An additional challenge, particularly for coastal systems (Grosholz & Ruiz, 2009), is the difficulty of defining a causal relationship in a dynamic system (Hale, Mavrogordato, Tolhurst, & Solan, 2014; Murray, Douglas, & Solan, 2014) because the expression of species contributions is context dependent (Godbold & Solan, 2009) and can take a long time to emerge (Godbold & Solan, 2013). Therefore, it becomes important to address when and how the occurrence of invasive species interacts with gradients of environmental variables that often characterize transitional habitats. These are seldom studied, especially in association with other aspects of directional forcing, such as, for example, aspects of climate change (but see Schneider, 2008; Weitere et al., 2009).

Here, we investigate how different size classes (a proxy for age) of a prominent invader of freshwater systems—the Asian Clam *Corbicula fluminea,* O.F. Müller, 1774, (Crespo et al., 2015; Sousa, Antunes, & Guilhermino, 2008)—affect important ecosystem processes (sediment fluid and particle transport) in the presence versus absence of warming across the freshwater–marine transition. *Corbicula fluminea* is known to affect hydrological processes, biogeochemical cycles, biotic interactions and the physical environment at an ecosystem scale (Sousa, Gutiérrez, & Aldridge, 2009; Sousa, Antunes, et al., 2008). Despite often being described as a freshwater bivalve, *C. fluminea* is an euryhaline species (salinity, up to 10–14; McMahon, 1983, 1999) and can colonize the areas upstream of estuaries (Franco et al., 2012; Ilarri, Souza, Antunes, Guilhermino, & Sousa, 2014; Sousa, Nogueira, Gaspar, Antunes, & Guilhermino, 2008). This is important because euryhaline species are able to invade along the freshwater‐marine continuum of the estuarine environment, presenting the possibility of temporary or permanent refugia that will allow longer term persistence (Crespo, Leston, Martinho, Pardal, & Dolbeth, 2017). Moreover, species that show phenotypic plasticity may be predisposed to establishing populations that are functionally dominant under climate change (e.g., Somero, 2010). By considering both the native freshwater habitat and the oligohaline waters of estuarine areas that have high invasive potential, we hypothesized that different sizes in macrobenthos could differentially mediate levels of ecological functioning and that temperature (in a simulated heat wave) could influence the intensity of the biological processes involved, with implications for long‐term functioning under a changing climate. We tested these ideas empirically by manipulating temperature, salinity, and *C. fluminea* size in a model marine benthic system.

## 2 METHODS

### 2.1 Sediment and fauna collection

Sediment and individuals of *C. fluminea* were collected in the oligohaline upper reaches of the Mondego Estuary, Portugal (40°9′47.91″N, 8°40′12.42″W) from a tidally influenced location. Sediment (gravel 38.7%, sand 58.9% and mud 2.5%, 0.4 ± 0.2% organic matter content, loss on ignition at 400°C, 8 hr) was defaunated using CO_2_‐enriched water (bubbled CO_2_) for ~1 hr to initiate upward movement of infaunal organisms to the sediment surface by lowering dissolved O_2_, before they were manually extracted using tweezers (adapted from Coelho, Flindt, Jensen, Lillebø, & Pardal, 2004).

### 2.2 Experimental set‐up and design

Our experimental design (see Figure S1) included all possible permutations of three different size classes of *C. fluminea*, with fixed biomass achieved through density adjustment (18.79 ± 0.94 g/aquaria wet biomass: *small*, measuring <1 cm, ~1 year old, 13 ind./aquaria (=902 ind/m^2^); *medium*, 2–2.5 cm, ~ 2 years old, 2 ind./aquaria^1^ (=138 ind./m^2^); *large, >*3 cm, >3 years old, 1 ind./aquaria^1^ (=69 ind/m^2^) at levels representative of the population at study site (Franco et al., 2012; Crespo et al. 2017, 2017). These size classes were crossed with two levels of salinity (freshwater, 0, and oligohaline, 5) and two levels of temperature (24 and 30°C) in glass aquaria (12 × 12 × 35 cm, internal dimensions). Each aquaria contained sediment (~10 cm depth) overlain with water to 30 cm depth. To minimize variation in habitat conditions, we used homogenized sediment and demineralized water, adding appropriate quantities of commercially available aquarium salt (Pro‐Reef, Tropic Marin®) to establish our oligohaline treatments. To distinguish the role of microbial and meiofaunal components of the system, we included a treatment where *C. fluminea* were absent. Temperature approximated summer water temperatures at the study site (24°C) or extreme heat‐wave conditions (30°C, Mouthon & Daufresne, 2006; Grilo, Cardoso, Dolbeth, Bordalo, & Pardal, 2011). Treatments representative of the natural habitat for *C. fluminea* (salinity, 0) contrasted to treatments (salinity, 5) representing either the estuarine gradient [e.g., 4.6 ± 3.1 in the mesohaline areas during flood events (Verdelhos, Cardoso, Dolbeth, & Pardal, 2014)] or areas of the estuary prone to drought events. Hence, our experimental design provides insight on how species contributions to ecosystem functioning are modified under those circumstances. Our experiment required a total of 48 aquaria (4 size treatments × 2 temperature × 2 salinity × 3 replicates, Figure S1). All aquaria were continually aerated and maintained under natural daylight conditions for a period of 6 days. Salinity, temperature, pH, and oxygen levels (O_2_) were measured at the beginning and at the end of the experiment. Realized experimental conditions are presented in Table S1).

### 2.3 Measurement of particle reworking (ecosystem process)

The extent of particle reworking—the passive and active displacement of sediment particles by the activity of macrofaunal organisms—was measured noninvasively using fluorescent sediment profile imaging (f‐SPI, Solan, Wigham, et al., 2004) after 6 days. Briefly, this method allows dyed sediment particles that fluoresce under UV light (luminophores: 30 g/aquaria, 125–250 μm diameter, green color; Brian Clegg, Ltd, UK), to be preferentially visualized (Schiffers, Teal, Travis, & Solan, 2011) and the distribution of luminophores to be determined at high spatial resolution from images of the side of the aquaria. We used a Canon EOS 350D single‐lens reflex digital CMOS camera (8.0 megapixels) set for an exposure of 10 s, diaphragm aperture diameter of *f* = 6.3, and a film speed (light sensitivity) equivalent to ISO 200. Images were saved in red‐green‐blue (RGB) color with JPEG (Joint Photographic Experts Group) compression, cropped to the full internal width of the aquaria (952 pixels, effective resolution = 126.1 μm per pixel) and analyzed using a custom‐made plugin that runs within ImageJ (Version 1.48c), a java‐based public domain program developed at the US National Institute of Health (available at http://imagej.nih.gov/ij/). Following Hale et al. (2014), we determined the mean (^f‐SPI^L_mean_, time‐dependent indication of mixing), median (^f‐SPI^L_med_, typical short‐term depth of mixing), and maximum (^f‐SPI^L_max_, maximum extent of mixing over the long term) mixed depth of particle redistribution. In addition, we determined the maximum vertical deviation of the sediment–water interface (upper − lower limit = surface boundary roughness, SBR), which provides an indication of surficial faunal activity.

### 2.4 Measurement of nutrient concentrations (ecosystem function)

Water samples (10 ml, prefiltered 0.45 μm, Whatman) were taken at day 0 before the introduction of fauna and at day 6 to determine water column nutrient concentrations (NH_3_‐N, NO_3_‐N, PO_4_‐P). These were analyzed with Continuous Flow Analyzer Skalar Sanplus with segmented flow analysis (SFA), using the Skalar methods: M461‐318 (EPA 353.2), M155‐008R (EPA 350.1), and M503‐555R (Standard Method 450‐P I).

### 2.5 Statistical analyses

We developed independent regression models for each of our dependent variables of particle reworking (SBR, ^f‐SPI^L_mean_, ^f‐SPI^L_med_, ^f‐SPI^L_max_) and nutrient concentrations (NH_3_‐N, NO_3_‐N, PO_4_‐P) using the full factorial combination of independent variables (*C. fluminea* size class, temperature, salinity). As our focus was to establish the effects of different size classes of *C. fluminea*, rather than presence versus absence effects, the procedural control (*C. fluminea* absent) was removed from the statistical analysis. As our data showed evidence of a violation of homogeneity, analyses were extended to include the appropriate variance covariate structure (minimal adequate model summaries are shown in the Supporting Information) using a generalized least squares (GLS) estimation procedure (Pinheiro & Bates, 2000). This procedure allows the residual spread to vary with the explanatory variables and avoids the need to transform data. For GLS, the optimal variance covariate structure was determined using restricted maximum‐likelihood (REML) estimation by comparing the initial regression model without a variance covariate structure to alternative regression models that include specific variance covariate structures using AIC and visual comparisons of model residuals. The optimal fixed structure was then determined by backward selection using the likelihood ratio (L‐ratio) test obtained using maximum‐likelihood (ML) estimation and the minimal adequate model was re‐expressed using REML (Diggle, Zeger, Liang, & Heagerty, 2002; West, Welch, & Gatecki, 2007; Zuur, Ieno, Walker, Saveliev, & Smith, 2009). As inferences about the relative importance of our explanatory variables, and their interactions, are based on the comparisons of the first level within each term with all other levels, we used a parametric bootstrap with 999 re‐samples and the percentile method to obtain the 95% CI limits around the predicted values (shown Supporting Information). All analyses were performed using the “R” statistical and programming environment (R Development Core Team 2012). GLS analyses were conducted using the “nlme” package (Pinheiro, Bates, DebRoy, & Sarkar, 2014) and parametric bootstrapping were conducted assuming that the estimated parameters followed a multivariate Gaussian distribution with mean and variances provided from the output of the fitting function, using the function “rmvnorm” within the package “mvtnorm” (Genz et al., 2014).

All data are available from Harvard Dataverse (Crespo, Solan, Leston, Pardal, & Dolbeth, 2017).

## 3 RESULTS

We found evidence that faunal activity and behavior, and associated nutrient concentrations, are affected by the size class of individuals of *C. fluminea*, temperature, and salinity (Table 1 and Models S1–S7), although the observed effects did not necessarily form full factorial interactions. Size class tended to be the most important variable, followed by temperature and/or salinity (least important). ANOVA confirmed that there were no differences amongst treatments at the start of the experimental period (day 0, *p*‐values > .05).

**Table 1:** Summary of significant terms found in the generalized least squares models, using bioturbation components and nutrient concentrations as dependent variables and size, temperature and salinity as explanatory variables

Dependent variable	Significant terms	*df*	L‐ratio	*p*
Bioturbation				
SBR	Size × temperature	5	17.323	.0039
^f‐SPI^L_mean_	Size × salinity	5	24.593	<.001
	Temperature	1	7.118	.0076
^f‐SPI^L_median_	Size × temperature	5	19.761	.0014
^f‐SPI^L_max_	Size	2	12.392	.002
Nutrients				
[NH_3_‐N]	Size × salinity	3	29.392	<.001
	Size × temperature	3	11.715	.0084
[NO_3_‐N]	Size	3	49.921	<.0001
	Salinity × temperature	3	18.854	<.001
[PO_4_‐P]	Size × temperature	3	15.499	.0014
	Size × salinity	3	14.541	.0023

### 3.1 Effects on ecosystem process

**Figure 1:**
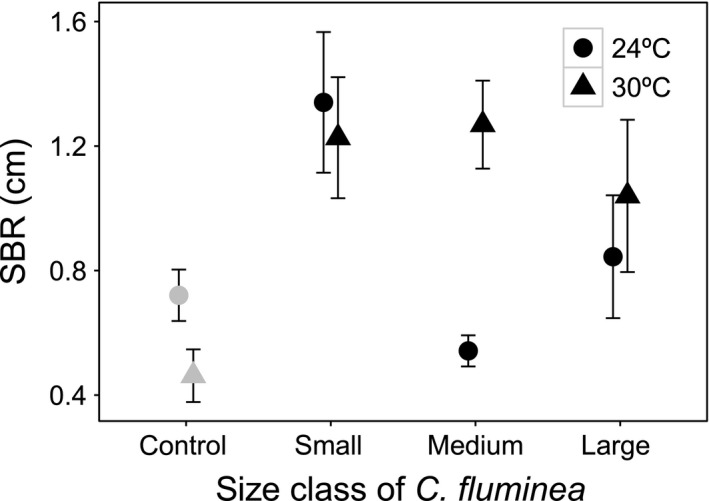
The interactive effects of *Corbicula fluminea* size class × temperature on surface boundary roughness (SBR: cm, mean ± *SE*). For clarity, jitter has been applied to the × = argument of the plot function to avoid overplotting. For comparison, SBR in the absence of *C. fluminea* is presented (gray, control)

^f‐SPI^L_mean_ was influenced by the interaction size class × salinity and an independent effect of temperature (Table 1, model structure described in Model S2). Size class, and all of its interactions, was the most influential variable (L‐ratio = 17.4282, *df* = 4, *p* = .0016), followed by salinity and its interactions (L‐ratio = 9.434963, *df* = 3, *p* = .024) and temperature (L‐ratio = 7.118, *df* = 1, *p* = .0076). ^f‐SPI^L_mean_ values ranged between 0.483 and 2.065 cm (small size class), to 0.159 and 0.851 cm (medium size class), and 0.288 and 1.476 cm (large size class). For both salinities, small‐sized individuals showed the highest values for ^f‐SPI^L_mean_, which was even higher at salinity 5 (mean ± *SE* (cm): salinity 0, 0.754 ± 0.097; salinity 5, 1.452 ± 0.191; Figure 2a). ^f‐SPI^L_mean_ values were smaller for medium‐sized individuals, but similar across salinity levels (mean ± *SE* (cm): salinity 0, 0.490 ± 0.099; salinity 5, 0.458 ± 0.096, Figure 2a). The ^f‐SPI^L_mean_ in the presence of larger individuals responded to increasing salinity (large sized, salinity 0 vs. salinity 5: *t*‐value = −2.376, *df* = 36, *p* = .0243; mean ± *SE* (cm): salinity 0, 0.530 ± 0.083; salinity 5, 0.676 ± 0.181, Figure 2a). ^f‐SPI^L_mean_ increased with increasing temperature (*t*‐value = 4.653, *df* = 36, *p* = .0001; mean ± *SE* (cm): 24°C, 0.704 ± 0.132; 30°C, 0.750 ± 0.081, Figure 2b).

Surface boundary roughness (SBR) was affected by a size class × temperature interaction, but not affected by salinity (Table 1, model structure described in Model S1). Temperature, and all of its interactions, was the most influential variable (L‐ratio = 13.497, *df* = 3, *p* = .0037), followed by size class and its interactions (L‐ratio = 11.197, *df* = 4, *p* = .0244). In the presence of *C. fluminea*, SBR ranged between 0.454 and 2.173 cm for the small individuals, to 0.454 and 1.853 cm for medium‐sized individuals, and 0.214 and 2.017 cm for the large‐sized individuals. At lower temperature, SBR reduced considerably at intermediate body size relative to populations of small and large individuals, but this trend was less compelling at the higher temperature (Figure 1). Small‐sized individuals tended to show higher SBR values, with similar values at both temperature regimes (mean ± *SE* (cm): 24°C, 1.340 ± 0.226; 30°C, 1.227 ± 0.194). The medium‐sized individuals showed a more pronounced effect of temperature, showing the smallest value of SBR (mean ± *SE*, cm) at 24°C (0.542 ± 0.050), which increased at 30°C (1.269 ± 0.141). The lowest SBR values tended to be found in large‐sized individuals, with slightly higher values at 30°C (mean ± *SE* (cm): 24°C, 0.845 ± 0.197; 30°C, 1.040 ± 0.245).

**Figure 2:**
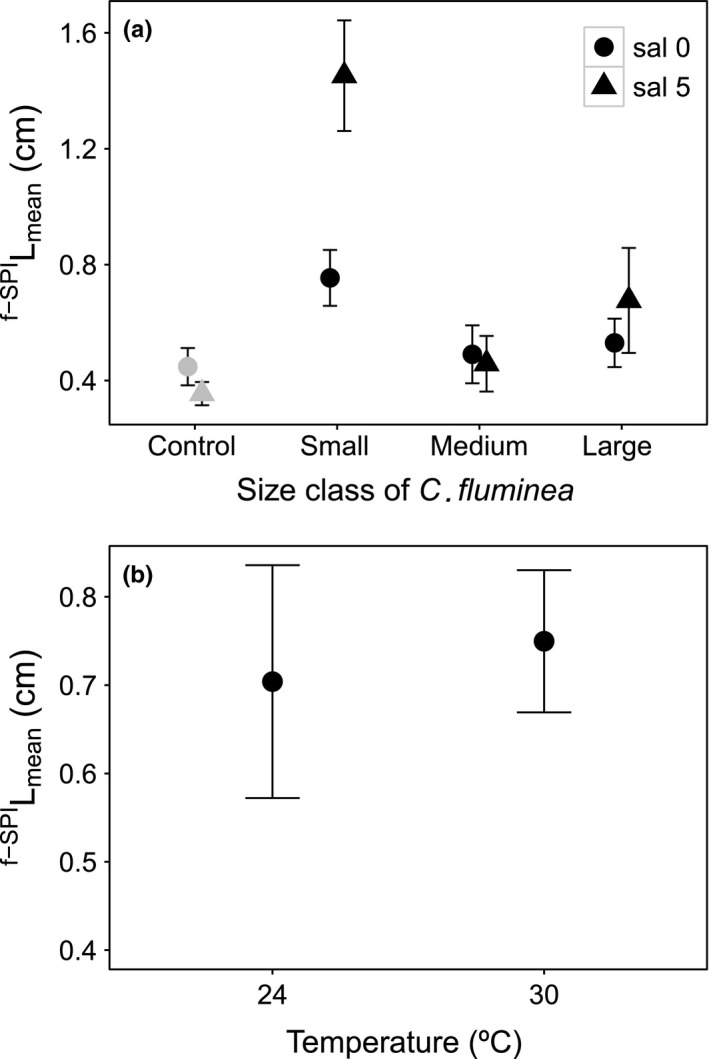
The interactive effects of *Corbicula fluminea* size × salinity (a) and the independent effect of temperature (b) on mean mixed depth of luminophores redistribution (^f‐^ ^SPI^L_mean_; cm, mean ± *SE*). For clarity, jitter has been applied to the × = argument of the plot function to avoid overplotting. For comparison, ^f‐^ ^SPI^L_mean_ in the absence of *C. fluminea* is presented (gray, control)

^f‐SPI^L_median_ was affected by the interaction size class × temperature, but unaffected by salinity (Table 1, model structure described in Model S3). Size class, and all of its interactions, was the most influential variable (L‐ratio = 18.377, *df* = 4, *p* = .001), followed by temperature and its interactions (L‐ratio = 14.846, *df* = 3, *p* = .002). ^f‐SPI^L_median_ values ranged between 0.315 and 2.395 cm (small size class), to 0.164 and 0.731 cm (medium size class) and 0.214 and 0.882 cm (large size class). Despite the small‐sized individuals showing the highest ^f‐SPI^L_median_ values at both temperatures, temperature effects seemed less important (mean ± *SE* (cm): 24°C, 1.071 ± 0.417; 30°C, 0.975 ± 0.181, Figure 3). Relative to small‐sized individuals, ^f‐SPI^L_median_ values decreased for medium and large size classes, at both temperatures. Interestingly, whilst ^f‐SPI^L_median_ increased with increasing temperature in medium‐sized individuals (mean ± *SE* (cm): 24°C, 0.258 ± 0.027; 30°C, 0.502 ± 0.062, Figure 3), the reverse was true for large‐sized individuals (mean ± *SE* (cm): from 24°C, 0.560 ± 0.095; 30°C, 0.398 ± 0.059, Figure 3).

**Figure 3:**
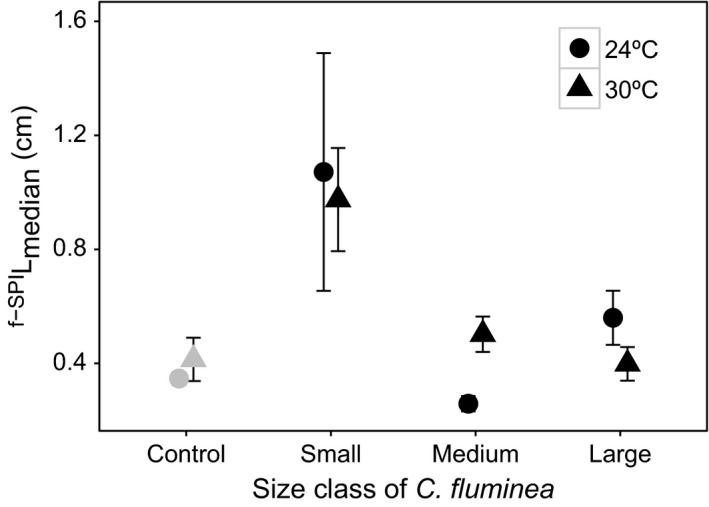
The interactive effects of *Corbicula fluminea* size class × temperature on median mixed depth of luminophores redistribution (^f‐^ ^SPI^L_median_; cm, mean ± *SE*). For clarity, jitter has been applied to the × = argument of the plot function to avoid overplotting. For comparison, ^f‐^ ^SPI^L_median_ in the absence of *C. fluminea* is presented (gray, control)

**Figure 4:**
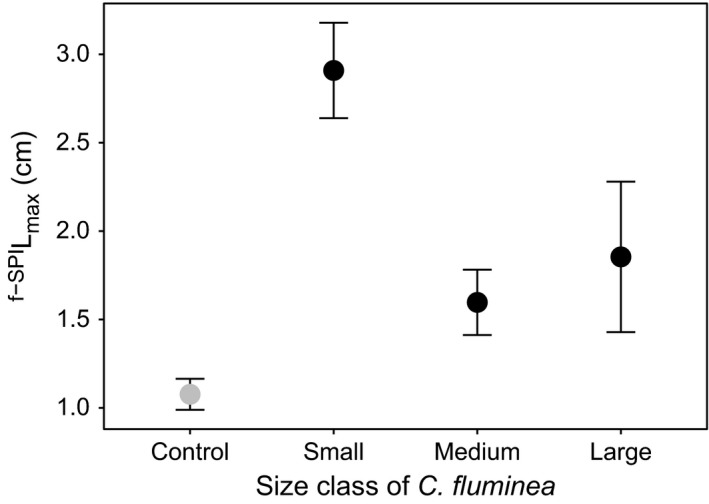
The independent effect of *Corbicula fluminea* size class on maximum mixed depth of luminophores redistribution (^f‐^ ^SPI^L_max_; cm, mean ± *SE*). For comparison, ^f‐^ ^SPI^L_max_ in the absence of *C. fluminea* is presented (gray, control)

For ^f‐SPI^L_max_, only size class was influential (Table 1, Model S4). Values ranged between 1.525 and 4.374 cm (small class size) to 0.756 and 2.609 cm (medium class size) and 0.681 and 5.206 cm (large class size). Mean values were highest for the small‐sized *C. corbicula* (mean ± *SE* (cm): 2.909 ± 0.270), followed by the large (mean ± *SE* (cm): 1.854 ± 0.425), and medium‐sized individuals (mean ± *SE*: 1.600 ± 0.185 cm) (Figure 4). ^f‐SPI^L_max_ for the small‐sized individuals was significantly different from medium and large sizes (*t*‐value = 4.012, *df* = 36, *p* = .0003 and *t*‐value = 2.093, *df* = 36, *p* = .0441, respectively), but there was no difference between medium and large size classes (*t*‐value = 0.555, *df* = 36, *p* = .5828).

### 3.2 Effects on nutrient concentration

**Figure 5:**
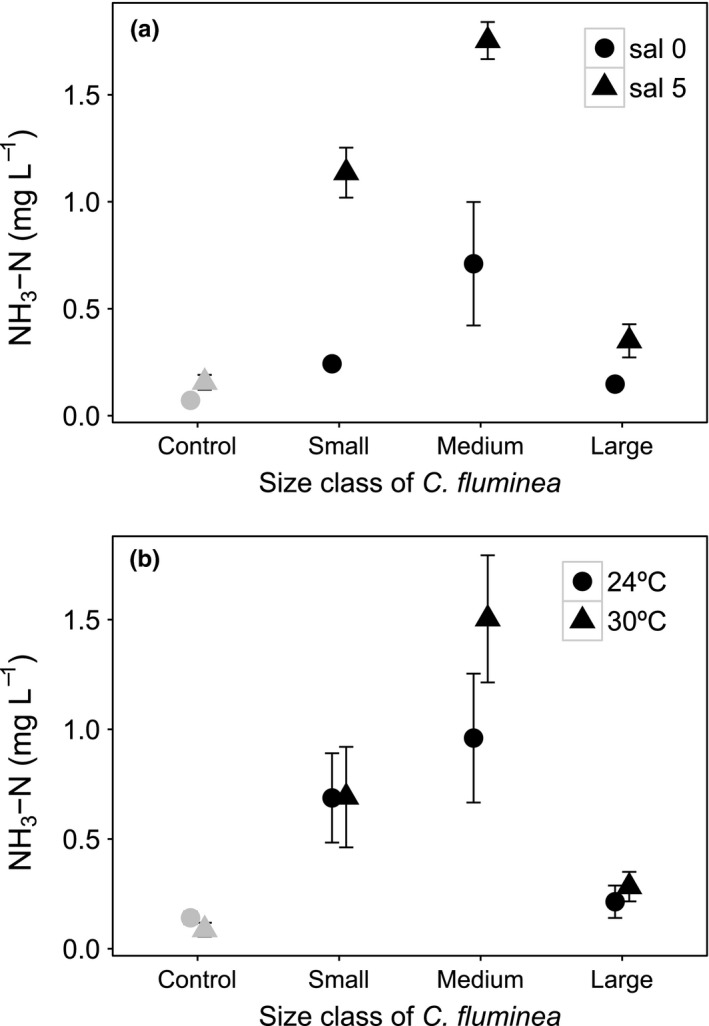
The interactive effects of *Corbicula fluminea* size × salinity (a) and *C. fluminea* size × temperature (b) on [NH_3_‐N] in the water (mg/L, mean ± *SE*). For clarity, jitter has been applied to the × = argument of the plot function to avoid overplotting. For comparison, [NH_3_‐N] in the absence of *C. fluminea* is presented (gray, control)

[NH_3_‐N] was dependent on size class × salinity and size × temperature interactions (Table 1, model structure described in Model S5). Size class and its interactions were more influential (L‐ratio = 57.236, *df* = 6, *p* < .0001) than salinity and its interactions (L‐ratio = 29.392, *df* = 3, *p* < .0001) and temperature and its interactions (L‐ratio = 11.715, *df* = 3, *p* = .0084). [NH_3_‐N] ranged from 0.200 to 1.543 mg/L in the presence of small‐sized individuals, from 0.118 to 1.933 mg/L in the presence of medium‐sized individuals and from 0.036 to 0.530 mg/L in the presence of large‐sized individuals of *C. fluminea*. [NH_3_‐N] increased with higher salinity when either medium‐sized (mean ± *SE*, mg/L: salinity 0, 0.710 ± 0.289; salinity 5, 1.754 ± 0.087, Figure 5a) or small‐sized individuals were present (mean ± *SE*, mg/L: salinity 0, 0.243 ± 0.017; salinity 5, 1.360 ± 0.117 cm, Figure 5a). The large‐sized individuals had the lowest [NH_3_‐N] for both salinity levels (mean ± *SE*, mg/L: salinity 0, 0.148 ± 0.019; salinity 5, 0.350 ± 0.078 cm, Figure 5a), similar to those when small‐sized individuals were present at salinity 0. Temperature had positive influence on [NH_3_‐N] when medium‐sized individuals were present (mean ± *SE*, mg/L: 24°C, 0.960 ± 0.294; 30°C, 1.504 ± 0.290 cm, Figure 5b), but was less influential when either small or large individuals were present, with the large‐sized clams showing the lowest [NH_3_‐N] (mean ± *SE*, mg/L: small size, 0.687 ± 0.203 at 24°C and 0.691 ± 0.229 at 30°C; large size, 0.214 ± 0.074 at 24°C and 0.283 ± 0.067 at 30°C, Figure 5b).

**Figure 6:**
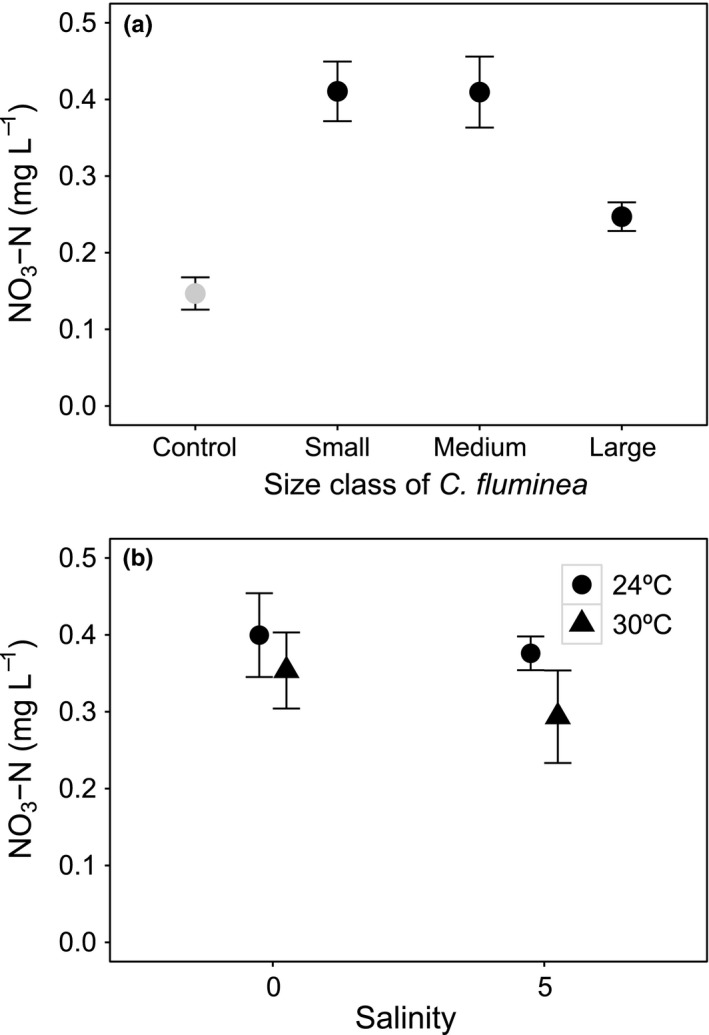
The independent effect of *Corbicula fluminea* size (a) and the interactive effects of *C. fluminea* size × temperature (b) on [NO_3_‐N] in the water (mg/L, mean ± *SE*). For clarity, jitter has been applied to the × = argument of the plot function to avoid overplotting. For comparison, [NO_3_‐N] in the absence of *C. fluminea* is presented (gray, control)

[NO_3_‐N] was affected by the interaction salinity × temperature and an independent effect of size (Table 1, model structure described in Model S6). Size was the most influential variable (L‐ratio = 49.921, *df* = 3, *p* < .0001), followed by temperature and its interactions (L‐ratio = 11.258, *df* = 2, *p* = .0036) and salinity and its interactions (L‐ratio = 7.999, *df* = 2, *p* = .0183). [NO_3_‐N] ranged from 0.173 to 0.659 mg/L when small‐sized individuals were present, from 0.137 to 0.732 mg/L when medium‐sized individuals were present, and from 0.158 to 0.31 mg/L when large‐sized individuals of *C. fluminea* were present. Small‐ and medium‐sized individuals showed similar values of [NO_3_‐N] (*t*‐value = 1.961, *df* = 36, *p* = .0592; mean ± *SE*, mg/L: 0.411 ± 0.039 and 0.410 ± 0.046, respectively, Figure 6a), which decreased for large‐sized individuals (*t*‐value = −4.582, *df* = 36, *p* = .0001; mean ± *SE*, mg/L: 0.247 ± 0.019, Figure 6a). A decrease in [NO_3_‐N] was shown with salinity 5, more accentuated at 30°C (Figure 6b). Also, in both salinity treatments, [NO_3_‐N] was lower at higher temperature (mean ± *SE*, mg/L: salinity 0, 0.400 ± 0.054, at 24°C and 0.354 ± 0.050, at 30°C; salinity 5, 0.376 ± 0.022, at 24°C and 0.294 ± 0.060, at 30°C).

**Figure 7:**
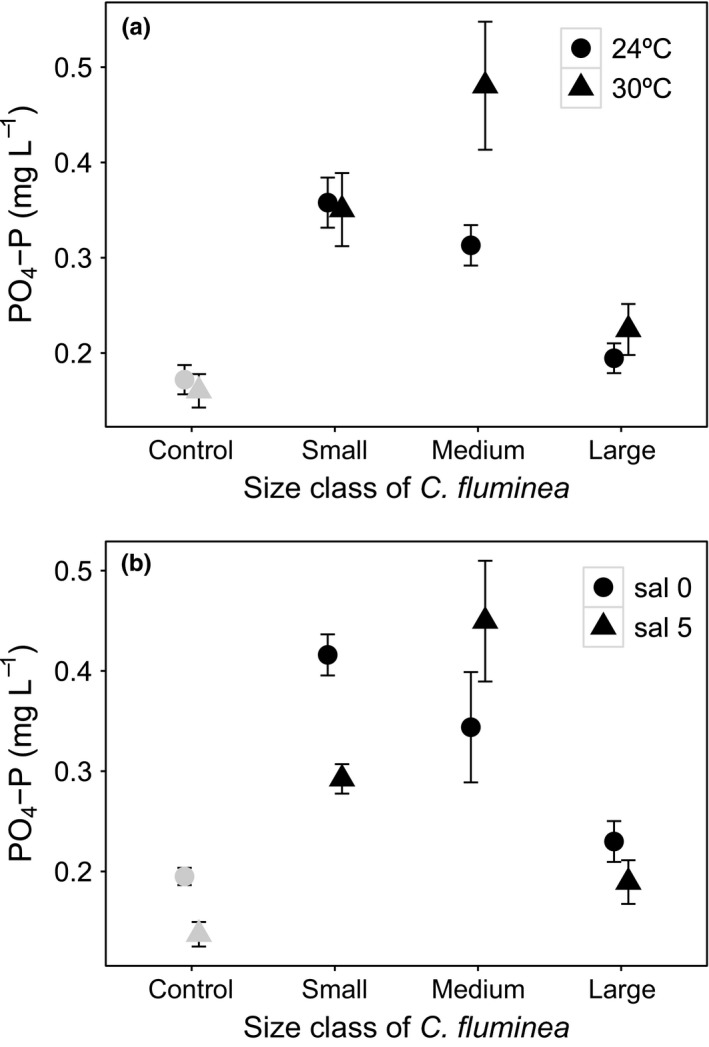
The interactive effects of *Corbicula fluminea* size × temperature (a) and *C. fluminea* size × salinity (b) on [PO_4_‐P] in the water (mg/L, mean ± *SE*). For clarity, jitter has been applied to the × = argument of the plot function to avoid overplotting. For comparison, [PO_4_‐P] in the absence of *C. fluminea* is presented (gray, control)

[PO_4_‐P] was influenced by the interactions size class × temperature and size class × salinity (Table 1, model structure in Model S7). Size, and its interactions, was the most influential variable (L‐ratio = 57.090, *df* = 6, *p* < .0001), followed by temperature and its interactions (L‐ratio = 15.499, *df* = 3, *p* = .0014) and salinity and its interactions (L‐ratio = 14.541, *df* = 3, *p* = .0023). [PO_4_‐P] ranged from 0.240 to 0.489 mg/L for small‐sized individuals, from 0.253 to 0.681 mg/L for medium‐sized individuals, and from 0.128 to 0.310 mg/L for large‐sized individuals of *C. fluminea*. Temperature tended to have a positive effect on [PO_4_‐P], particularly when medium‐sized individuals were present (mean ± *SE*, mg/L: 0.313 ± 0.021 at 24°C and 0.481 ± 0.067 at 30°C, Figure 7a). [PO_4_‐P] was similar at both temperatures when small‐sized individuals were present (*t‐*value = −0.296, *df* = 36, *p* = .77; mean ± *SE*, mg/L: 0.358 ± 0.026 at 24°C and 0.351 ± 0.038 at 30°C, Figure 7a), and also similar between temperatures when large‐sized individuals were present (*t*‐value = 0.413, *df* = 36, *p* = .683; mean ± *SE*, mg/L: 0.195 ± 0.016 at 24°C and 0.225 ± 0.027 at 30°C, Figure 7a). For salinity, there was a tendency for [PO_4_‐P] to decrease in the presence of small‐ and large‐sized individuals, whilst the reverse was true when medium‐sized individuals were present (mean ± *SE*, mg/L: 0.416 ± 0.020 for small size, 0.344 ± 0.055 for medium size and 0.230 ± 0.020 for large size, Figure 7b).

## 4 DISCUSSION

When the ecological impacts of invasive species have been characterized, there is a tendency to assume that previously observed effects are uniformly expressed across novel circumstances (Powell, Chase, & Knight, 2013). Our findings suggest that this assertion is not necessarily appropriate, however, as the mediation of important ecosystem functions vary with the size of the organism and with environmental context (here, temperature, and salinity). Interestingly, despite strong temperature forcing across a gradient of salinity, we found a consistently prominent effect of size class on ecosystem functioning throughout all of our response variables. Whilst size class is an important trait underpinning bioturbation processes (Norkko, Villnäs, Norkko, Valanko, & Pilditch, 2013; Solan, Cardinale, et al., 2004), it was not necessarily the most important predictor of associated nutrient dynamics (Séguin, Harvey, Archambault, Nozais, & Gravel, 2014). Although we did not measure physiological condition directly, larger body size is known to minimize species vulnerability to the cyclic nature of physico‐chemical conditions (Gardner, Peters, Kearney, Joseph, & Heinsohn, 2011), which is particularly important across the freshwater–estuarine transition (Crespo, Leston, et al., 2017).

The effects of body size we documented highlight the importance of species population structure for the ecosystem functioning, but our study also highlights the importance of species‐environment interactions and the role on abiotic and biotic context. Small‐sized individuals contributed the most for bioturbation, presumably because smaller individuals may face less mechanical resistance (de la Huz, Lastra, & López, 2002) and are also more responsive to changes in environmental conditions (Gardner et al., 2011; Godbold, Bulling, & Solan, 2011; Werner & Gilliam, 1984). In addition, small individuals of *C. fluminea* have been reported to have higher metabolic requirements (Xiao et al., 2014) and, as they invest more in tissue growth than larger‐sized individuals, increased particle mixing may lead to increased feeding effort. Mattice and Dye (1976) determined the lower and upper lethal temperatures of *C. fluminea* as 2 and 34.8°C, respectively, which spans our tested range. Larger individuals of *C. fluminea* seem to be less affected by temperature changes (as reported elsewhere for other bivalves, *Mytilus edulis* and *M. leucophaeata*, Rajagopal, van der Velde, van der Gaag, & Jenner, 2005), which may explain why large *C. fluminea* maintain similar levels of behavior across different temperature treatments. In contrast to the present study, however, Majdi, Bardon, and Gilbert (2014) tested the effects of body size on bioturbation behavior in *C. fluminea* with a similar approach and found that medium and large sizes contributed more to bioturbation at temperatures closer to the mid‐tolerance range of the species. We are unable to rule out an effect of body size in the present study, but acknowledge that density‐related differences in body size could be responsible for a synergistic effect on particle remobilization and resuspension. As size‐class proportions used in our study are closely related to those occurring in the original stock (Crespo, Leston, et al., 2017; Franco et al., 2012), our findings are, nevertheless, relevant to the natural system. Similarly, the effects of salinity also increased the medium mixing depth of small‐sized individuals, perhaps a response to increasing water salinity whilst maintaining pedal feeding on the surface. Nevertheless, salinity seems to play a less important role in moderating the mediation of ecosystem properties by *C. fluminea*. Xiao et al. (2014) found that narrow salinity ranges had small effect on metabolism of *C. fluminea*. However, other mechanisms, such as reducing valve opening time and/or physiological responses to salinity (Dietz, Wilcox, Byrne, Lynn, & Silverman, 1996; McCorkle & Dietz, 1980; Ruiz & Souza, 2008), are likely to be more effective that mechanical displacement over extended periods of time. An increase in salinity associated with a period of drought, or in relation to the natural dispersion of *C. fluminea,* is unlikely to radically shift species behavior. As an osmoconformer, *C. fluminea* individuals increase extracellular ionic concentrations and reduce intracellular volume to cope with hyperosmotic stress (McCorkle & Dietz, 1980; Ruiz & Souza, 2008). Evans, Murphy, Britton, and Newland (1977) found that *C. fluminea* shows different responses (and tolerance) to salinity, depending on the geographic origin of the population stock or historical acclimation status. As older individuals are larger and will have been pre‐exposed to salinity changes, this explanation is consistent with the effects of body size and may explain the smaller impact of salinity on bioturbation/particle reworking mediated by larger individuals.

Irrespective of the mechanisms involved, the most important finding of our study is that some species that are capable of invading multiple habitats (such as *C. fluminea*), once established, modify ecosystem properties in ways that reflect the environmental conditions of the locality. However, the relationship between bioturbation intensity and nutrient generation is difficult to predict based on trait values alone, especially as the organism–sediment interactions alter with context (Hale et al., 2014; Murray et al., 2014; Teal, Parker, & Solan, 2010). We cannot discount the role of salinity and temperature in influencing meiofaunal and microbial communities. For instance, we observed a reduction in NO_3_ and an increase in NH_3_ with increasing temperature. Changes in temperature and salinity are known to influence dissimilatory nitrate reduction to ammonium (DNRA) (Giblin, Weston, Banta, Tucker, & Hopkinson, 2010; Giblin et al., 2013), which implies that the reduction in nitrate to ammonium either by fermentative or autotrophic DNRA is enhanced under estuarine conditions (Bonaglia, Nascimento, Bartoli, Klawonn, & Brüchert, 2014; Koop‐Jakobsen & Giblin, 2010; Sousa et al., 2012). Simultaneously, anaerobic oxidation of ammonium will occur, but the importance of this pathway, at least in coastal and estuarine sediments, is reduced when compared to DNRA (Bonaglia et al., 2014; Giblin et al., 2013; Gilbertson, Solan, & Prosser, 2012). In addition, individuals will also contribute to nutrient release via excretion, which may be of greater relative importance than bioturbation activity at a certain threshold of body size. Certainly, larger individuals have a larger siphon and exhibit lower rates of particle mixing as they do not need to relocate to exploit food resources (Zwarts, Blomert, Spaak, & de Vries, 1994). For large‐sized *C. fluminea*, grazing on primary producers and removing particulates and sorbed phosphates might, at least in part, explain the observations for PO_4_ (Phelps, 1994). Small‐ and medium‐sized individuals of *C. fluminea* were responsible for greater particle reworking, which could stimulate meiofauna (via increasing aeration and solute availability) and the release of PO_4_ at the sediment–water interface (Piot, Nozais, & Archambault, 2014). Whilst all of these explanations are feasible, however, there is still a lot of uncertainty regarding the mechanistic basis by which species alter the functioning of an ecosystem, despite the well‐known effects of NIS on the structure of communities and biodiversity (e.g., Ilarri et al., 2014; Phelps, 1994). This is particularly concerning, given that biological invasions are expected to rise and ecological niches may become favorable for invasive species under climate change (Crespo, Leston, et al., 2017; Gama, Crespo, Dolbeth, & Anastácio, 2016; Montoya & Raffaelli, 2010).

The results we present here may represent a realistic outcome for a natural system under a full invasion, with a monotonous community consisting of only *C. fluminea*. Our findings confirm the possibility that non‐native species that can access and thrive in multiple environmental conditions along the freshwater–marine transition can have dramatic effects on ecosystem properties. Whilst these effects can vary with environmental context, they are likely to manifest at larger scales and across perceived environmental boundaries. A challenge for the management of such transitional habitats will be in determining whether residual populations, perhaps occupying suboptimal conditions, can re‐establish more widely. Source–sink dynamics must be accounted in management efforts, particularly in highly variable environments, such as estuaries. Within the freshwater–marine transition, demographic surplus from population sources may provide new colonization opportunities in sink habitats (Heinrichs et al., 2016), where local reproduction is low or not possible, as evidenced in, for example, Crespo, Leston, et al, (2017). If so, control measures will need to recognize that perceived environmental constraints may be an insufficient means of prioritizing the application of control measures and that distinguishing species as freshwater versus marine may be inadequate.

## CONFLICT OF INTEREST

The authors declare no conflict of interest.

## AUTHOR CONTRIBUTIONS

M.S., M.P., and M.D. conceived the ideas; D.C, S.L, M.P., and M.D. collected the data; D.C. M.S., and M.D. analyzed the data; D.C. led the writing.
