# Supplementary material for: Ecological consequences of invasion across the freshwater–marine transition in a warming world
Source: Ecol Evol. 2018 Jan 11;8(3):1807–17. doi: 10.1002/ece3.3652 (PMC5792526; doi:10.1002/ece3.3652)
Supplement: Supplementary file 1 [file ECE3-8-1807-s001.docx]

**SUPPORTING INFORMATION**

**
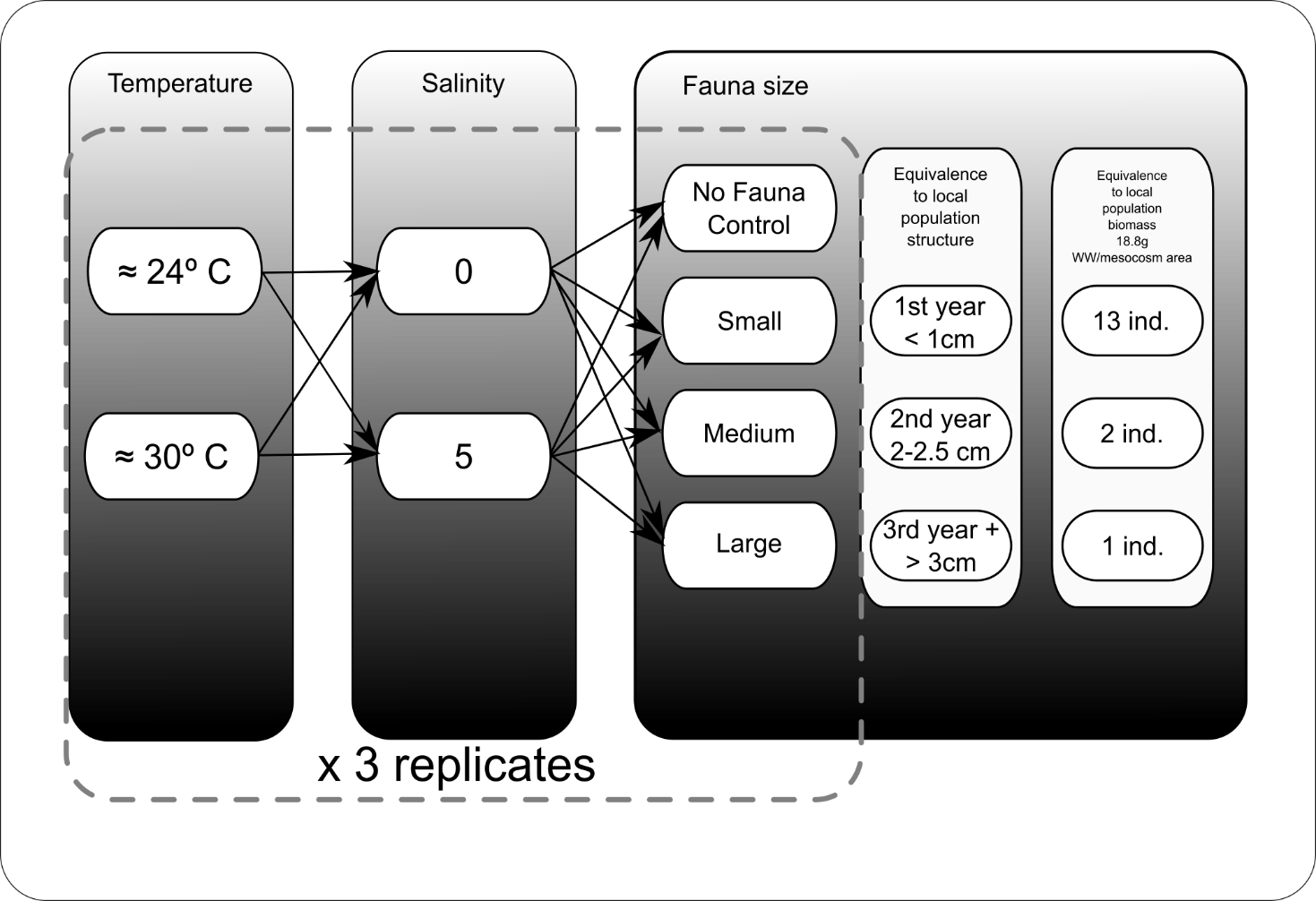
Figure S1** Experimental design: the experiment included all possible permutations of three different size classes of *Corbicula fluminea* (small, medium and large sizes) exposed to two levels of salinity and two levels of temperature (WW: wet weight).

| **Table S1** Realised experimental conditions for all treatments | | | | |
| --- | --- | --- | --- | --- |
| Factor levels | Salinity  (mean ± standard deviation, PSU) | Temperature  (mean ± standard deviation, ºC) | Dissolved oxygen  (mean ± standard deviation, mg.L^-1^; %) | pH |
| “Salinity 0” | 0 |  |  | 8.9 ± 0.53 |
| “Salinity 5” | 4.4 ± 0.17 |  |  | 8.9 ± 0.53 |
| “24ºC” |  | 24.0 ± 1.6 | 8.0 ± 0.4; 95 ± 2.2 | 8.9 ± 0.53 |
| “30ºC” |  | 29.7 ± 1.2 | 6.4 ± 0.6; 85 ± 5.1 | 8.9 ± 0.53 |

**Structure of the minimal adequate models, with body size, temperature and salinity as explanatory variables**

***Bioturbation***

**Model S1** *Surface Boundary Roughness (SBR)*

SBR = f (size + temperature + size x temperature)

The model was a linear regression model with a GLS extension, with size as variance covariate.

| **Table S2** Minimal adequate model coefficient estimations for SBR | | | | |
| --- | --- | --- | --- | --- |
|  | Value | S.E. | t-value | p-value |
| Intercept (size_1_;temp_24_) | 1.340 | 0.211 | 6.360 | 0.0000 |
| size_2_ | -0.798 | 0.243 | -3.292 | 0.0026 |
| size_3_ | -0.496 | 0.306 | -1.619 | 0.1163 |
| temp_30_ | -0.113 | 0.298 | -0.381 | 0.7062 |
| size_2_ x temp_30_ | 0.840 | 0.339 | 2.475 | 0.0194 |
| size_3_ x temp_30_ | 0.309 | 0.433 | 0.713 | 0.4816 |

**Figure S2** Predictions of the minimal adequate regression model (GLS extension with *size* as variance covariate) for the Surface Boundary Roughness for varying clam size class at two levels of temperature: horizontal bars represent the predicted values from the minimal adequate regression model (n = 36) and the vertical lines represent the confidence intervals (± 95 %).**Model S2** *Mean Luminophore Depth (^f-SPI^ L_mean_)*

^f-SPI^ L_mean_ = f (size + salinity + temperature + size x salinity )

The model was a linear regression model with a GLS extension, with size x salinity x temperature as variance covariate.

| **Table S3** Minimal adequate model coefficient estimations for ^f-SPI^ L_mean_ | | | | |
| --- | --- | --- | --- | --- |
|  | Value | S.E. | t-value | p-value |
| Intercept (size_1_;temp_24;_sal_0_) | 0.614 | 0.082 | 7.481 | 0.0000 |
| size_2_ | -0.216 | 0.091 | -2.386 | 0.0238 |
| size_3_ | -0.146 | 0.086 | -1.712 | 0.0975 |
| sal_5_ | 0.423 | 0.155 | 2.728 | 0.0107 |
| temp_30_ | 0.301 | 0.065 | 4.653 | 0.0001 |
| size_2_ x sal_5_ | -0.513 | 0.182 | -2.817 | 0.0086 |
| size_3_ x sal_5_ | -0.720 | 0.188 | -3.842 | 0.0006 |

**Figure S3** Predictions of the minimal adequate regression model (GLS extension with *size x salinity x temperature* as variance covariate) for ^f-SPI^L_mean_ for varying clam size class at two levels of salinity and temperature: horizontal bars represent the predicted values from the minimal adequate regression model (n = 36) and the vertical lines represent the confidence intervals (± 95 %).

**Model S3** *Median Luminophore Depth (^f-SPI^ L_median_)*

^f-SPI^ L_median_ = f (size + temperature + size x temperature)

The model was a linear regression model with a GLS extension, with size x salinity as variance covariate.

| **Table S4** Minimal adequate model coefficient estimations for ^f-SPI^ L_med_ | | | | |
| --- | --- | --- | --- | --- |
|  | Value | S.E. | t-value | p-value |
| Intercept (size_1_;temp_24_) | 0.484 | 0.146 | 3.318 | 0.0024 |
| size_2_ | -0.229 | 0.152 | -1.509 | 0.1417 |
| size_3_ | -0.020 | 0.153 | -0.130 | 0.8974 |
| temp_30_ | 0.315 | 0.206 | 1.528 | 0.1369 |
| size_2_ x temp_30_ | -0.082 | 0.214 | -0.382 | 0.7052 |
| size_3_ x temp_30_ | -0.370 | 0.216 | -1.713 | 0.0970 |

**Figure S4** Predictions of the minimal adequate regression model (GLS extension with *size x salinity* as variance covariate) for ^f-SPI^L_med_ for varying clam size class at two levels of temperature: horizontal bars represent the predicted values from the minimal adequate regression model (n = 36) and the vertical lines represent the confidence intervals (± 95 %).

**Model S4** *Maximum Luminophore Depth (^f-SPI^ L_max_)*

^f-SPI^ L_max_ = f (size)

The model was a linear regression model with a GLS extension, with size as variance covariate.

| **Table S5** Minimal adequate model coefficient estimations for ^f-SPI^ L_max_ | | | | |
| --- | --- | --- | --- | --- |
|  | Value | S.E. | t-value | p-value |
| Intercept (size_1_) | 2.909 | 0.270 | 10.779 | 0.0000 |
| size_2_ | -1.312 | 0.327 | -4.012 | 0.0003 |
| size_3_ | -1.055 | 0.504 | -2.093 | 0.0441 |

**Figure S5** Predictions of the minimal adequate regression model (GLS extension with *size* as variance covariate) for ^f-SPI^L_max_ for varying clam size class: horizontal bars represent the predicted values from the minimal adequate regression model (n = 36) and the vertical lines represent the confidence intervals (± 95 %).

***Nutrients***

**Model S5** *NH_3_-N concentration*

NH_3_-N concentration = f (size + salinity + temperature + size x salinity + size x temperature)

The model was a linear regression model with a GLS extension, with size x salinity as variance covariate.

| **Table S6** Minimal adequate model coefficient estimations for NH_3_-N concentrations | | | | |
| --- | --- | --- | --- | --- |
|  | Value | S.E. | t-value | p-value |
| Intercept (size_1_;temp_24;_sal_0_) | 0.247 | 0.027 | 9.136 | 0.0000 |
| size_2_ | 0.293 | 0.259 | 1.129 | 0.2688 |
| size_3_ | -0.126 | 0.036 | -3.538 | 0.0015 |
| sal_5_ | 0.894 | 0.119 | 7.513 | 0.0000 |
| temp_30_ | -0.009 | 0.038 | -0.236 | 0.8156 |
| size_2_ x sal_5_ | -0.015 | 0.284 | 0.527 | 0.6023 |
| size_3_ x sal_5_ | -0.691 | 0.142 | -4.867 | 0.0000 |
| size_2_ x temp_30_ | 0.350 | 0.113 | 3.090 | 0.0046 |
| size_3_ x temp_30_ | 0.062 | 0.050 | 1.236 | 0.2272 |

**Figure S6** Predictions of the minimal adequate regression model (GLS extension with *size* x *salinity* as variance covariate) for NH_3_-N concentration for varying clam size class at two levels of salinity and temperature: horizontal bars represent the predicted values from the minimal adequate regression model (n = 36) and the vertical lines represent the confidence intervals (± 95 %).**Model S6** *NO_3_-N concentration*

NO_3_-N concentration = f (size + salinity + temperature + salinity x temperature)

The model was a linear regression model with a GLS extension, with size x salinity as variance covariate.

| **Table S7** Minimal adequate model coefficient estimations for NO_3_-N concentrations | | | | |
| --- | --- | --- | --- | --- |
|  | Value | S.E. | t-value | p-value |
| Intercept (size_1_;temp_24;_sal_0_) | 0.356 | 0.035 | 10.157 | 0.0000 |
| size_2_ | 0.083 | 0.042 | 1.961 | 0.0592 |
| size_3_ | -0.127 | 0.028 | -4.582 | 0.0001 |
| sal_5_ | 0.080 | 0.034 | 2.342 | 0.0260 |
| temp_30_ | 0.012 | 0.032 | 0.376 | 0.7099 |
| sal_5_ x temp_30_ | -0.123 | 0.043 | -2.858 | 0.0077 |

**Figure S7** Predictions of the minimal adequate regression model (GLS extension with *size x salinity* as variance covariate) for NO_3_-N concentration for varying clam size class at two levels of salinity and temperature: horizontal bars represent the predicted values from the minimal adequate regression model (n = 36) and the vertical lines represent the confidence intervals (± 95 %).

**Model S7** *PO_4_-P concentration*

PO_4_-P concentration = f (size + salinity + temperature + size x salinity + size x temperature)

The model was a linear regression model with a GLS extension, with salinity x temperature as variance covariate.

| **Table S8** Minimal adequate model coefficient estimations for PO_4_-P concentrations | | | | |
| --- | --- | --- | --- | --- |
|  | Value | S.E. | t-value | p-value |
| Intercept (size_1_;temp_24;_sal_0_) | 0.412 | 0.013 | 32.902 | 0.0000 |
| size_2_ | -0.123 | 0.018 | -6.964 | 0.0000 |
| size_3_ | -0.217 | 0.018 | -12.241 | 0.0000 |
| sal_5_ | -0.112 | 0.032 | -3.496 | 0.0017 |
| temp_30_ | -0.012 | 0.042 | -0.296 | 0.7698 |
| size_2_ x sal_5_ | 0.175 | 0.045 | 3.868 | 0.0006 |
| size_3_ x sal_5_ | 0.101 | 0.045 | 2.230 | 0.0342 |
| size_2_ x temp_30_ | 0.198 | 0.060 | 3.322 | 0.0026 |
| size_3_ x temp_30_ | 0.030 | 0.060 | 0.501 | 0.6203 |

**Figure S8** Predictions of the minimal adequate regression model (GLS extension with *size x temperature* as variance covariate) for PO_4_-P concentration for varying clam size class at two levels of salinity and temperature: horizontal bars represent the predicted values from the minimal adequate regression model (n = 36) and the vertical lines represent the confidence intervals (± 95 %).
